# The importance of including habitat-specific behaviour in models of butterfly movement

**DOI:** 10.1007/s00442-020-04638-4

**Published:** 2020-04-06

**Authors:** Luke C. Evans, Richard M. Sibly, Pernille Thorbek, Ian Sims, Tom H. Oliver, Richard J. Walters

**Affiliations:** 1 School of Biological Sciences, University of Reading, Whiteknights, PO Box 217, Reading, Berkshire RG6 6AH UK; 2 Syngenta, Jealott’s Hill International Research Centre, Bracknell, Berkshire RG42 6EY UK; 3 Centre for Environmental and Climate Research, University of Lund, Lund, Sweden; 4 BASF SE, APD/EE, Speyerer Strasse 2, 67117 Limburgerhof, Germany

**Keywords:** Motivation, Habitat fragmentation, Individual-based model, Dispersal kernel, Leptokurtosis, *Maniola jurtina*

## Abstract

Dispersal is a key process affecting population persistence and major factors affecting dispersal rates are the amounts, connectedness and properties of habitats in landscapes. We present new data on the butterfly *Maniola jurtina* in flower-rich and flower-poor habitats that demonstrates how movement and behaviour differ between sexes and habitat types, and how this effects consequent dispersal rates. Females had higher flight speeds than males, but their total time in flight was four times less. The effect of habitat type was strong for both sexes, flight speeds were ~ 2.5 × and ~ 1.7 × faster on resource-poor habitats for males and females, respectively, and flights were approximately 50% longer. With few exceptions females oviposited in the mown grass habitat, likely because growing grass offers better food for emerging caterpillars, but they foraged in the resource-rich habitat. It seems that females faced a trade-off between ovipositing without foraging in the mown grass or foraging without ovipositing where flowers were abundant. We show that taking account of habitat-dependent differences in activity, here categorised as flight or non-flight, is crucial to obtaining good fits of an individual-based model to observed movement. An important implication of this finding is that incorporating habitat-specific activity budgets is likely necessary for predicting longer-term dispersal in heterogeneous habitats, as habitat-specific behaviour substantially influences the mean (> 30% difference) and kurtosis (1.4 × difference) of dispersal kernels. The presented IBMs provide a simple method to explicitly incorporate known activity and movement rates when predicting dispersal in changing and heterogeneous landscapes.

**Electronic supplementary material:**

The online version of this article (10.1007/s00442-020-04638-4) contains supplementary material, which is available to authorized users.

## Introduction

Movement is a key feature of animal behaviour that provides insight into a species behaviour and ecology, and which ultimately affects the persistence and stability of populations in fragmented and heterogeneous landscapes (Hanski 1998; Tscharntke et al. 2012; Bonte and Dahirel 2017). Fragmented habitats are widespread as a result of human activities (Kerr and Deguise 2004; Corvalan et al. 2005), and representing species movements across heterogeneous landscapes is required to forecast the responses of populations to increasing anthropogenic pressure (Fahrig and Merriam 1994; Lima and Zollner 1996). Accurate movement models need to take account of both the movement capacity of individuals and their behaviour (Morales and Ellner 2002; Kokko and López-Sepulcre 2006; Nathan et al. 2008). This is a problem well suited to individual-based models that include behavioural changes occurring in responses to different resources and/or motivations, as demonstrated for beetles (Morales and Ellner 2002), elephant seals (Bestley et al. 2012), caribou (Mason and Fortin 2017), turtles (Jonsen et al. 2006), and also butterflies (Brown and Crone 2016b).

Butterflies are a useful model system to investigate the mechanisms influencing dispersal rate (Stevens et al. 2010) because their movement can be tracked in the field (Root and Kareiva 1984; Schultz et al. 2012) and their behaviour observed simultaneously (Dennis 1992). They also show rapid responses to environmental change and thus can act as indicators species for assessing community health (Thomas 2005; Rákosy and Schmitt 2011). In recent years, many UK species have declined in either abundance or distribution (Fox et al. 2015) and understanding the mechanistic links between habitat change and butterfly movement may be of increasing importance for conservation. A common framework has developed for measuring and describing butterfly movement (Root and Kareiva 1984; Turchin 1991; Schultz and Crone 2001) and the effect of habitat on movement has been well demonstrated for many species, suggesting that the behavioural responses to varying habitat are quite general (Odendaal et al. 1989; Fownes and Roland 2002; Schtickzelle et al. 2007)*.* Aspects of these effects have been summarised for the meadow brown butterfly (*Maniola jurtina* L.) by Delattre et al. (2010), who showed that both activity and movement are variable between habitat transitions, a phenomenon attributed to a foraging or dispersal ‘mood’. We suggest *M. jurtina* is a suitable general system for further evaluating the effects of habitat in butterfly movement models as it is widespread, characteristic of grassland species (Van Swaay et al. 2013), and lives in metapopulations with dispersal rates comparable to many other species (Conradt et al. 2000, 2001; Schneider et al. 2003a; Dapporto et al. 2011). It is also thought to have undergone moderate declines as a result of habitat loss (Van Swaay et al. 2013; Fox et al. 2015), though more recent estimates suggest population stability at the European scale (Van Swaay et al. 2019).

Butterfly movement models have demonstrated the importance of individual movement for a range of processes such as meta-population dynamics (Ovaskainen and Hanski 2004; Heinz et al. 2006), home ranges sizes (Hovestadt and Nowicki 2008; Kőrösi et al. 2008), functional connectivity (Ovaskainen et al. 2008a), and minimum area requirements (Brown and Crone 2016a). These models typically rely on simulating movement as correlated random walks (Turchin 1991), or diffusion approximations of this process, which consist of a sequence of discrete ‘steps’ and ‘turns’ which approximate the flight path in simulation. A limitation of this approach is that it ignores, or simplifies, activity budgets so that time spent stationary or duration of flights are only very roughly approximated. This may limit our understanding of the effects of motivation, activity or resources density on movement rates as they may differ between substantially between habitats and sexes (Reim et al. 2019). The effects of changing behaviour in movement models can have strong effects on their predictions (Lima and Zollner 1996; Morales and Ellner 2002; Pauli et al. 2013) and habitat-dependent changes in movement rates have been quantified for only a small minority of species (Zalucki and Kitching 1982; Odendaal et al. 1989; Roland et al. 2000; Fownes and Roland 2002; Schtickzelle et al. 2007; Ovaskainen et al. 2008b; Schultz et al. 2019). Further, the additional benefit of explicit representation of activity budgets, such as duration of flight and periods of inactivity within individual-based models (IBMs), has been recently demonstrated by explaining variation in intraspecific dispersal rate (Brown and Crone 2016b) and the responses of butterflies to changing weather conditions (Evans et al. 2019a). Though a simple innovation, the inclusion of activity budgets in movement models may have additional consequences for forecasting dispersal in heterogeneous landscapes and these are yet to be fully explored.

Our study had two complementary aims: (1) to quantify the changes in behavioural time budgets and movement rates of *M. jurtina* across two different habitat types; and, (2) to explore the consequences for forecasting dispersal of including activity budgets in individual-based models of movement in complex landscapes. Activity budgets here are simplified behavioural time budgets in which activities are categorised as either flight or non-flight. We show that taking account of habitat-dependent differences in activity is crucial to obtaining good fits of an individual-based model to movement data and that this has important implications for forecasting longer-term dispersal in heterogeneous habitats.

## Material and methods

### Study species and sites

The meadow brown butterfly (*M. jurtina*) is a common butterfly found across a variety of grasslands in the British Isles (Brakefield 1982a). The larvae feed predominantly on *Poa* spp., though also on other grasses and common herbs (Ouin et al. 2008). The adults obtain nectar from a range of flowers common to grasslands (Dennis 1992) favouring knapweeds (*Centaurea* spp.) and thistles (*Cirsium* spp.) (Brakefield 1982a; Lebeau et al. 2017). The species is univoltine with a broad flight period, typically between June and September (Thomas 2010), and the species exhibits protandry with the males emerging earlier than females (Scali 1971; Brakefield 1982b). The females are monandrous and mate quickly after emergence (Dowdeswell 1981), though males may mate more than once and spend time searching for females (Brakefield 1982a). Females appear relatively unselective for host plants when egg laying (Delattre et al. 2010) though show a preference for shorter grasses and herbs (Lebeau et al. 2015). Adult life span has been measured to be between 5 and 12 days, though it can be more than 20 (Brakefield 1982b), with survival likely dependent on the amount and quality of nectar resources (Lebeau et al. 2016; Evans et al. 2019b).

Data were collected in June and July of 2018 at four grassland areas proximate to the University of Reading (51° 26′ N, 0° 56′ W). Two, labelled here as resource poor, were mown short turf grasslands with minimal flowering plants, the other two areas, labelled resource rich, were meadow grasslands containing a variety of grass species and wildflowers predominantly the common knapweed (*Centaurea nigra,* L.) with small amounts of other common grasslands species such as spear thistle (*Cirsium vulgare*, Savi). The patches were generated by conservation-friendly management, where areas of grassland (approximately 60 × 100 m) had been left fallow to support biodiversity. We provide a map of a study site in the supplementary materials (Fig. S4).

### Movement and behavioural observations

200 (♀100, ♂100) individuals were followed for a maximum of 10 min (mean 352.4 s, range 11.4–603.7 s) with movement and behaviour recorded simultaneously. Butterflies were opportunistically followed between the hours of 9:00 and 13:00 with flight paths collected as a series of steps and turns (Turchin 1991). Observers maintained a distance of approximately 3 m from the butterfly and coordinated to record the position and time every time the butterfly landed, with a marker flag placed in the ground, or after 15 s if the butterfly did not land. This observation distance has been shown to have no impact flight behaviour (Root and Kareiva 1984) and following opportunistically assures butterflies were engaged in normal behaviour prior to observation. After three observations in one patch, observers switched to the other. This was done so that the behaviours of butterflies were approximately balanced for the time of day, weather effects, and also as it reduced the chance of following the same butterflies. As this precaution was taken, and butterflies were numerous, the probability of following the same butterfly was considered low. Flight paths were recorded up to a maximum of 15 flags at which point the observation was stopped. Observations were stopped prematurely if butterflies left the habitat in which the observation started or if during the observation an individual’s identity was uncertain due to the presence of, or interaction with, conspecifics. Stopping due to interaction was rare and not expected to bias the results. 10 min and 15 flags were selected to capture useful data on butterfly movement behaviour while also collecting a representative number of individuals. The location of flags was subsequently mapped using a high-grade Global Navigation Satellite System receiver (Arrow 200 RTK).

For the analysis, five main flight statistics were recorded: step speed was calculated as (distance between successive flags)/(time taken), *turning angle* as the angle subtended between successive steps, flight duration as the time between the beginning and the end of the flight, inter-flight duration as the total time in between flights, and displacement as the Euclidean distance between the start and end locations of the observation. Step speed and turning angles were used as measures of movement rate and flight duration and inter-flight duration of activity (inter-flight containing all non-flight behaviour). ‘Movement rate’ here is used to refer to parameters describing the directedness and speed of flight, and ‘activity’ refers to the amount of time in flying or stationary. Thus, we draw a distinction between the way the butterfly moves during flight and the total amount of time in flight. During the observations, behaviours were recorded continuously by categorising behaviour into flying, nectaring (taking nectar from flowers), basking (open wings and stationary), inactive (closed wing and stationary), or ovipositing (Dover 1989). Timing of behaviour was recorded accurately using a bespoke android phone app developed for the project by LE.

### Statistical analysis

Linear mixed-effect models were used for inference of the effect of sex and habitat type, as fixed factors, on both activity budget and movement components. To control for repeated measures from an individual, means of the movement metrics calculated for each 10-min observation period were used as the dependent variables. Additionally, to control for variation between sampling days, a random intercept for the day of observation was introduced into all models. Model diagnostics were used to check the conformation of the data to the assumptions of linear models and minimal transformations were used when residuals were skewed. Thus, log transformations were used for step speed, flight durations, inter-flight durations, and short-term displacement rate. Models were fitted using the R package ‘lme4’ (Bates et al. 2015), *p* values were obtained using the Satterthwaite approximation for degrees of freedom using the R package ‘LmerTest’ (Kuznetsova et al. 2017), and conditional and marginal *R*^2^ (Nakagawa and Schielzeth 2013) were calculated using the ‘MuMin’ package (Barton 2019). As multiple tests were conducted on the same dataset, a Bonferroni correction was applied to the *p* values obtained across all the linear models. Wallraff rank-sum tests of angular distance were used to test for differences in turning angles between sexes and habitat types using the circular package in R (Agostinelli and Lund 2017) and a Bonferroni correction was again applied to the *p* values to control for multiple testing between groups. The analysis of the behavioural time budget data required compositional analysis, as engaging in one behaviour (e.g. basking) precludes the possibility of engaging in any other. To test for differences between sexes and habitats for time engaging in each of the behaviours, Dirichlet regression was performed using the R package ‘DirichletReg’ (Maier 2014). As oviposition was relatively infrequent, it was analysed separately, and logistic regression was used to predict oviposition occurring in the two habitat types at any point during observations. Bonferroni corrections were applied to *p* values derived from analysis of the time budget. All analyses were conducted in R (R Core Team 2019).

### Individual-based model

**Fig. 1:**
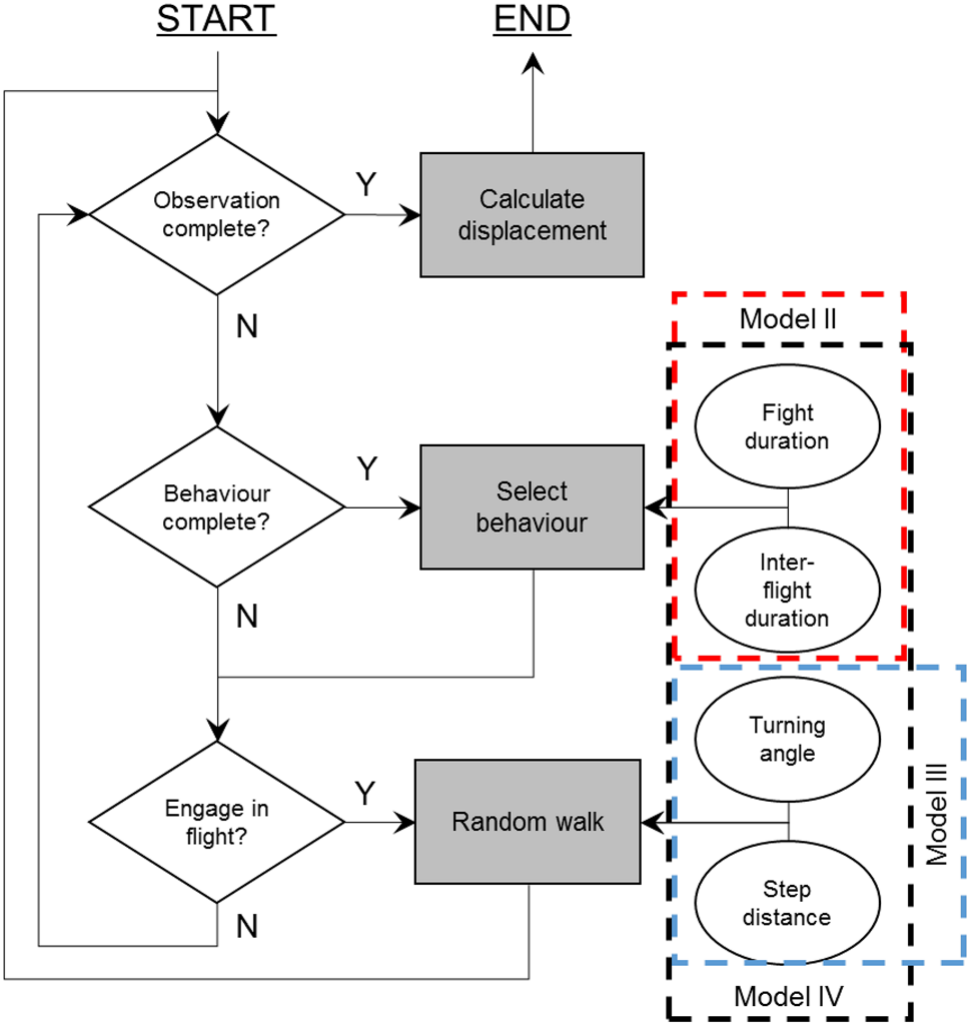
Conceptual model of the IBM. Squares represent model processes, diamonds decision points, and circles input into the model. Dashed lines represent parameterisations in which the enclosed data are habitat specific (methods), and model I with no habitat-specific parameters is omitted. The model runs on time steps of 1 s

A spatially explicit individual-based random walk model was developed to evaluate the effect of habitat heterogeneity on the movements and activity budget of *M. jurtina*. The model consists of individuals representing butterflies that move across a grid of habitat patches. Individuals do not die or reproduce. The model is conceptually similar to that used by Brown and Crone (2016a) containing an addition to the more standard correlated random walk approaches, with the movement explicitly represented as transitions between flights and inter-flight periods. An overview of the model is as follows (Fig. 1): first, random draws from the distributions of flight and inter-flight durations are imported into the model. The individuals select an inter-flight duration and remain stationary until this time has elapsed. Next, the individuals draw a flight duration. To move during a flight, the individuals draw step distances from marginal distributions of step lengths observed for flights of that duration. For example, if a 4-s flight was drawn, a corresponding step from the 4-s marginal distribution of step lengths would be selected. The butterfly then moves forward at a rate such that the step length is completed in the flight time. As step lengths were measured at a maximum of every 15 s, a long flight may result in multiple steps being drawn before the flight time has elapsed. This detail, which is not included in standard random walk approaches, decouples movement rate from flight time and is important here to accurately approximate the effect of changing flight durations on displacement. After a flight, or every 15 s during flight, the individuals change heading by drawing a turning angle and adding this turn to the current heading. After the flight time has elapsed, the individuals select another inter-flight duration and this process repeats until the end of the simulation. Distributions for the habitat-specific flight and inter-flight durations were produced by interpolations on the empirical cumulative distribution functions fitted to the data. The model was built in NetLogo 6.0 (Wilensky 1999) and analysis was carried out using the RNetLogo package (Thiele 2014). von Mises circular distributions were fitted to observed turning angles using the ‘circular’ package in R (Agostinelli and Lund 2017). Full description of the IBM is provided using the Overview, design concepts and detail (ODD) protocol in the supplementary materials (Grimm et al. 2010).

The effect of habitat type on movement and activity, and their subsequent effects on short-term net displacement (the 10-min observation time of the field methods), was investigated by contrasting four model versions of the IBM. In each model, parameter values for movement rates and activity budgets were drawn from empirically derived distributions for each sex separately. To evaluate the effect of habitat-dependent movement, we began with a null model (model I), in which neither movement rate nor activity changed as habitat changed, so parameter values were drawn from distributions without regard to movement rate or activity. Model II evaluates what happens if activity budget is habitat specific, so activity parameter values were habitat specific. In model III, movement rate parameter values were habitat specific, and in model IV all parameter values were habitat specific. To evaluate the accuracy of the predictions from the four models, we compared the mean short-term net displacement from the field observations to those predicted by the models. To measure differences between the observed and predicted, we used the root mean squared error (RMSE), which gives the average mismatch between observed and predicted in the original units (metres).

To investigate the implications of model structure for longer-term displacement, models I, III and IV were used to predict dispersal kernels over varying habitat compositions. Dispersal was measured as the Euclidean displacement over the simulation time. We selected the models on the grounds that model I represented a null model, model III corresponded to a standard habitat-specific correlated random walk, and model IV is the total effect of habitat-specific activity and movement. Model parameter values were drawn from either habitat-specific or pooled distributions for each sex and movement trait or activity. To generate realistic landscapes an area of 3 km^2^ was simulated using a fractal landscape algorithm (Saupe 1988; Jackson and Fahrig 2012) with the Hurst parameter, which controls the clumping of resource-rich habitat patches in the fractal landscape, and the total proportion of resource-rich habitat over adjusted between simulations. 5000 butterflies were initialised, and simulations were run for 5 days of simulated time with eight virtual hours of flight time per day. An initial burn-in of eight virtual hours was used to remove the effect of randomization of the butterflies’ starting locations before movements were recorded. Data on simulated butterfly movement recorded during the simulation was used for subsequent analyses.

## Results

The study had two goals: to quantify the effect of habitat differences on movement rates and behaviour and to evaluate the inclusion on these effects in an IBM.

**Fig. 2:**
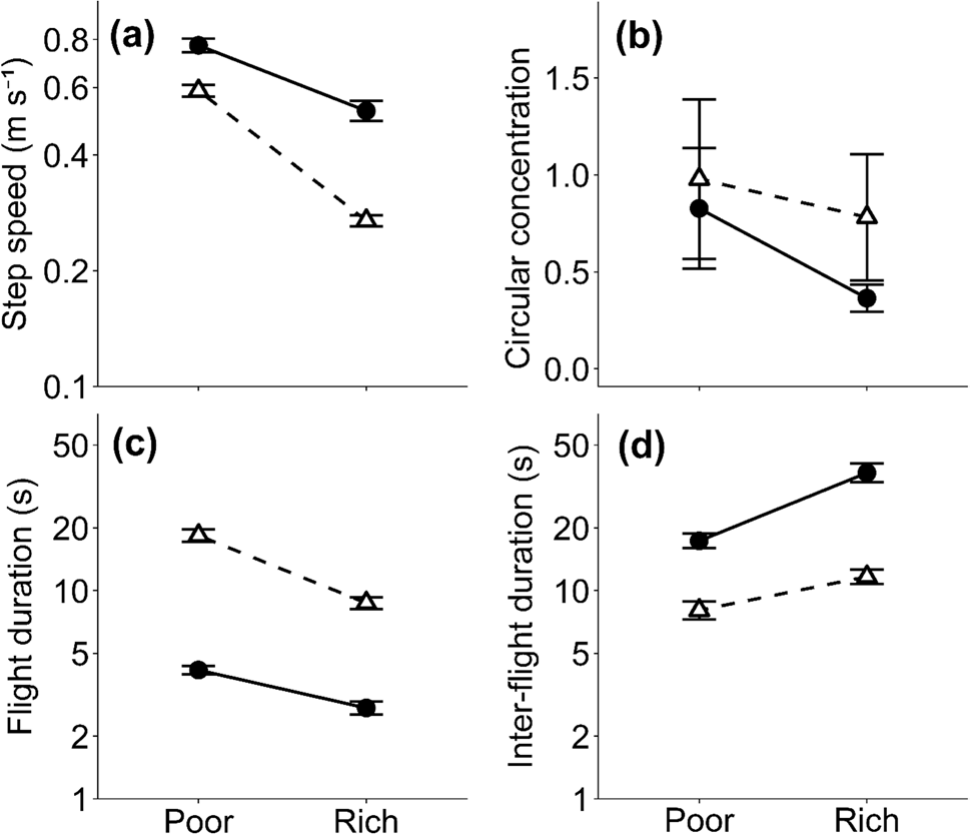
Effect of habitat and sex of *M. jurtina* on **a** step speed; **b** straightness of flight, measured as circular concentration; **c** flight durations; **d** inter-flight durations. Males shown as open triangles, and females as solid circles. Note log scales are used to stabilise variances in **a;c,d** bars show standard errors

With regard to movement rates on resource-rich and resource-poor areas, on resource-poor areas both sexes flew faster (Fig. 2a) and straighter (Fig. 2b), and performed longer flights (Fig. 2c) with shorter intervals between flights (Fig. 2d) (for all comparisons, supplementary material Table 1 and 2). The resultant short-term net displacement was three times greater on resource-poor areas than on resource-rich areas (Fig. 4). Comparing the sexes, female flights were faster than males, but they were shorter, less straight in the resource-poor habitat, and the intervals between flights were also longer so the resultant displacement rates of females were less than those of males (*p* < 0.01 for all comparisons, supplementary material Table 1).

**Fig. 3:**
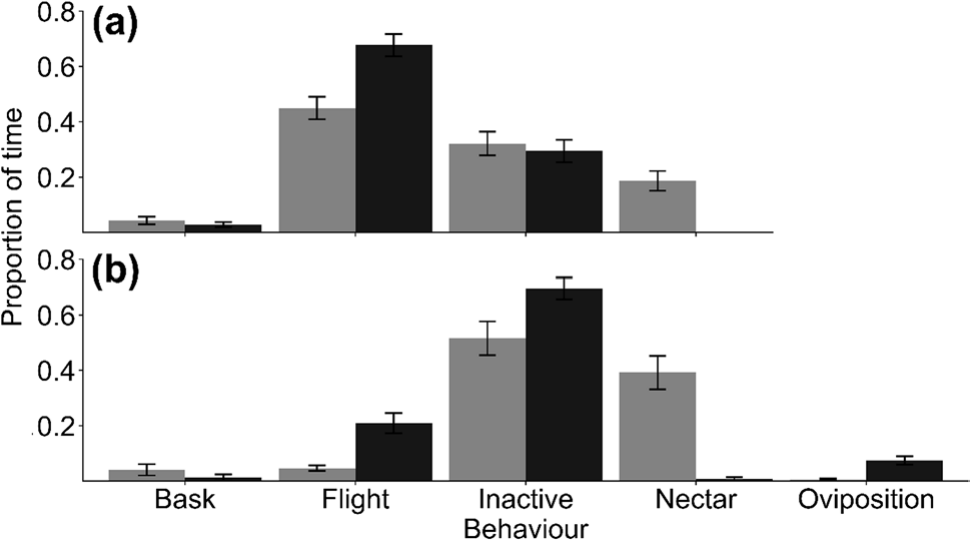
Behavioural time budgets for **a** males; and **b** females. Grey bars show resource rich, and black bars resource poor. Bars show standard errors

With regard to behavioural time budgets, in the resource-rich habitat females spent about 40% of their time nectaring and less than 10% of their time flying, with the rest of the time spent mostly inactive (50%) rather than actively basking (Fig. 3). In contrast, on the resource-poor habitat females spent more of their time flying (20%) and otherwise inactive (~ 70%); however, this was where we observed the ovipositing (10%). Males, by contrast, spent most of their time flying, ~ 65% on resource-poor and ~ 45% on resource-rich areas, and were only inactive for about 30% of their time (Fig. 3), but like females, they spent more time nectaring in the resource-rich areas. Full details of statistical testing are given in supplementary material Table 3.

**Fig. 4:**
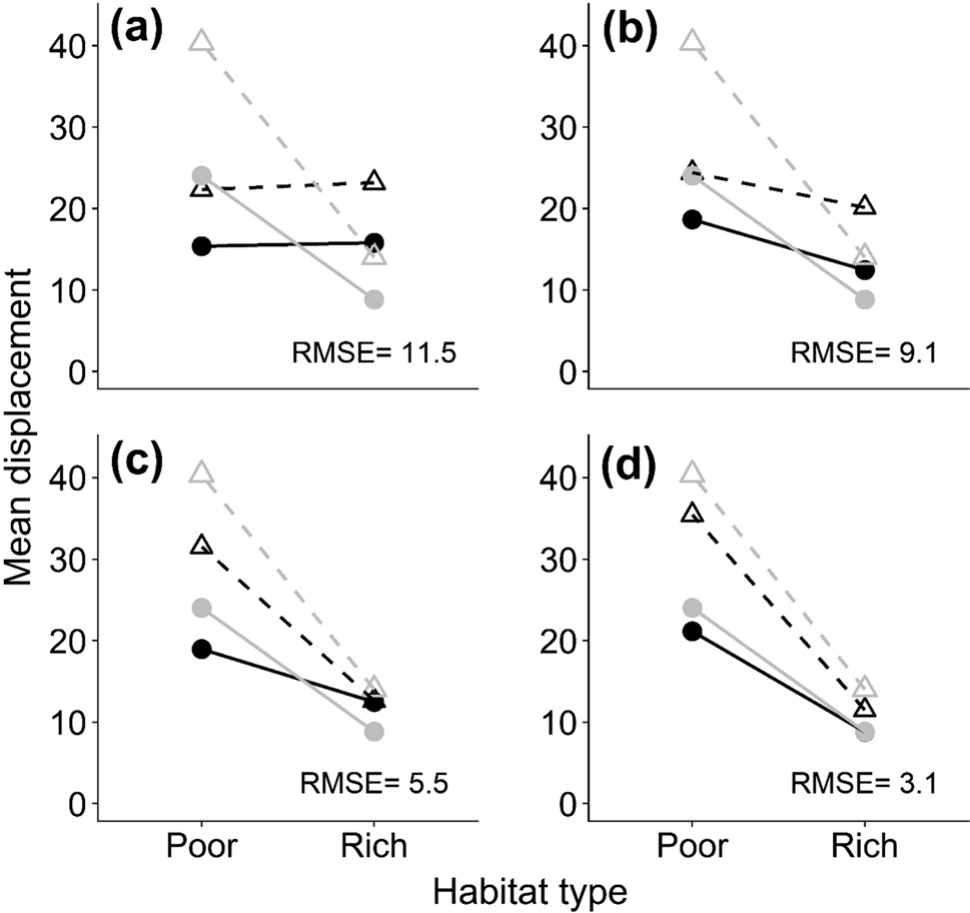
Observed and modelled short-term displacements, observed in grey, model predictions in black. **a** Model I, neither movement rates nor behaviours take account of habitat; **b** model II, behaviours take account of habitat; **c** model III, movement rates take account of habitat; **d** model IV, both movement rates and behaviours are habitat specific. Males shown as open triangles, and females as solid circles

To evaluate the joint effects of habitat and model structure on short-term net displacement, we compared the predictions of the four IBM models described in “Materials and methods”. In the first model, neither movement nor activity was habitat specific. In the second, activity was habitat specific; in the third, movement was habitat specific; and in the fourth, both activity and movement were habitat specific. Prediction of short-term net displacement was poor if no allowance was made for habitat (Fig. 4a, RMSE = 11.5), but improved if activity (Fig. 4b, RMSE = 9.1) or movement (Fig. 4c, RMSE = 5.5) parameters were habitat-specific. Prediction of displacement was best if both activity and movement rate were habitat specific (Fig. 4d, RMSE = 3.1).

**Fig. 5:**
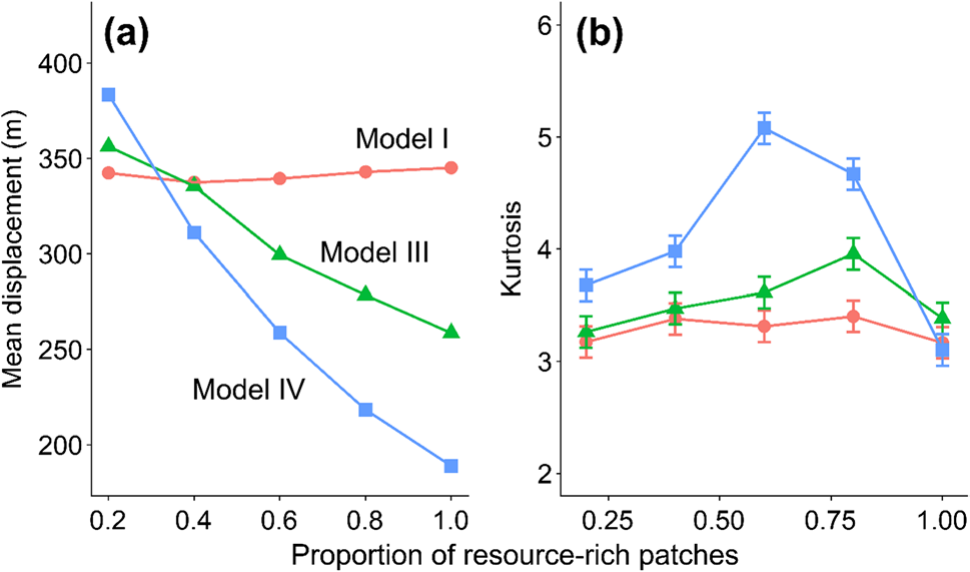
The effect of model structure on 5-day displacement statistics for female butterflies: **a** mean displacement; **b** kurtosis. Model I represents a null model, without habitat-specific movement and behaviour; model III represents the standard movement model with habitat-specific steps and turns and model IV represents the full movement model, where both movement rules and motivation to move are all habitat specific. 95% confidence intervals for kurtosis were estimated as ± 0.14 using standard methods (Wright and Herrington 2011)

To investigate the implications of model structure for longer-term displacement, we ran the models in fractal landscapes (see “Materials and methods”) differing in the proportion of the landscape, and aggregation of habitat that was resource rich, with striking results (Figs. 5, S1, S2). For clarity, we present the effects of the proportion of landscape for an intermediate fixed level of aggregation for females, effects for males, and results across all habitat configurations in Figs. S1 and S2.

Model structure influenced longer-term displacement predictions (Fig. 5a). Whereas using model I (red points, no model structure) displacement is unaffected by the proportion of resource-rich patches, the more structured models III and IV show increasing negative effects. In model IV, displacement at maximum resource-patch density is half that when only 20% of patches are resource rich, and is ~ 30% lower than model III. The effects on kurtosis—measuring the disproportionate contribution to dispersal of far-moving individuals, are also substantial (Fig. 5b). At a resource-patch proportion of 60%, kurtosis under model IV approaches ~ 1.6 × its value under model I and ~ 1.4 × its value under model III.

## Discussion

Our objective in this paper has been to quantify the influence of habitat type on the activity and movement rate of *M. jurtina* and to integrate these effects into an IBM. We found that both the activity and the movement rate of butterflies differed substantially between the habitat types. Including the changes in activity, in addition to habitat-specific movement rate, within the model structure, improved accuracy when predicting short-term displacement and had large consequences for the mean and shape of the resultant dispersal kernel over longer time periods.

Our findings that *M. jurtina* flew faster in resource-poor areas than in resource-rich areas (Fig. 2) are similar to the effects found in other butterfly species (Zalucki and Kitching 1982; Odendaal et al. 1989; Roland et al. 2000; Fownes and Roland 2002; Schtickzelle et al. 2007; Ovaskainen et al. 2008b) and we attribute these differences to adaptive responses to nectar flower densities (Brakefield 1982a). Habitat-specific movement rules such as these are quite general across taxa and likely emerge from foraging strategies adapted to trade-offs between efficient resource detection (Smith 1974; Haskell 1997) and predation risk (Zollner and Lima 2005). In insects, slower flight speeds and increases in tortuosity could be expected to improve detection and assessment of potential resources on the wing (Chittka et al. 2003), while faster, straighter movements could reduce time and energy spent in unfavourable and unproductive habitats (Bartumeus et al. 2008). The differences between the sexes—males flights were straighter and lasted longer with shorter inter-flight durations—likely reflect differences in the motivation for movement. When not searching for nectar plants, males may be primarily engaged in searching for females, a behaviour termed ‘patrolling’ (Brakefield 1982a; Shreeve 1984), whereas females spend time locating suitable egg-laying sites, or inactive to avoid the unwanted attentions of males post-copulation. Sex-specific motivations are, therefore, consequential for understanding butterfly movement, as the purpose of flight and the probability of its initiation are often different between the sexes. That females flew faster than males (Fig. 2a) is expected from first-principles scaling given the larger size of females (Norberg and Rayner 1987; Dudley and Srygley 1994), though their total movement is less due to greater inactivity.

With few exceptions, females oviposited in the mown grass habitat, but foraged in the resource-rich habitat (Fig. 3). *M. jurtina*’s preference for short grasses has been previously noted (Dennis 1992; Lebeau et al. 2015). The general reasons for the preferences of butterflies for host plants have been quite well explored (Thomas et al. 2011; Dennis 2012). In general, oviposition sites are chosen to maximise nutritional quality (Warren 1984; Dennis 1985; Pullin 1987; Ravenscroft 1994) and/or the suitability of the microclimate (Dennis 1983; Thomas 1983, 2010; Thomas et al. 1986). Consequently, suitable larval resources are often a small subset of the total host plant cover. That mown grass will have new growth and will be warmer than long grass may be reasons why this habitat was favoured for ovipositing. Another possible reason for the selection of the mown grass is that butterflies are generally at higher densities around nectar resources (Brakefield 1982a), which increases the probability of harassment from males (Odendaal et al. 1989) with negative consequences for females fitness (Turlure and Van Dyck 2009).

It, therefore, seems that females faced a trade-off between ovipositing without foraging in the mown grass, or foraging without ovipositing where flowers were abundant. This likely affects the distribution of butterflies observed in a landscape. Lebeau et al. (2015) noted that after grass strips were mown at an arable site, the abundance of female *M. jurtina* increased fourfold, while that of males changed little, and variation in micro-distribution of the sexes has been noted for *M. jurtina* and other butterfly species (Brakefield 1982a; Odendaal et al. 1989). For species with spatial segregation of host and nectar plants, this trade-off between resources has been seen to have important effects on population dynamics (Fred et al. 2006). Consequently, such effects are likely relevant for conservation management, as well-adopted agri-environment schemes, such as nectar-rich field margins (Vickery et al. 2009), may offer only part of the desired resources for even a generalist species like *M. jurtina*. Therefore, a mix of habitat types, i.e. complementation (Dunning et al. 1992), is likely beneficial for butterflies (Schultz and Dlugosch 1999; Ouin et al. 2004) and likely other taxa.

Deployment of the IBM shows that taking account of habitat-dependent differences in activity is crucial to obtaining good fits of an individual-based model to movement data (Fig. 4). Habitat-dependent movement rates have so far only be quantified for a minority of species hampering conservation management efforts (Schultz et al. 2019) and those containing changing activity levels are even fewer. IBMs, such as presented here, provide a useful platform to improve predictions of movement across complex landscapes. Our relatively simple IBM included only the additional effects of changing flight and inter-flight durations (Brown and Crone 2016b), but these had strong effects on the mean and shape of predicted dispersal kernels across varying habitat configurations (Fig. 5). These effects are important because the level of kurtosis reflects the relative proportion of individuals dispersing long distances. Dispersal kernels that are highly leptokurtic (kurtosis > 3), otherwise known as ‘fat-tailed’, have important consequences for rate of range expansion, gene flow and consequently population genetic structure across the species range (Ibrahim et al. 1996; Nathan et al. 2012). Here, we demonstrated that maximum kurtosis occurs when there are similar amounts of resource-poor and -rich habitat (Fig. 5b, blue points). This can be explained by bearing in mind that kurtosis reflects the variation between individuals in how far they disperse. When the landscape is composed of equal amounts of the two habitats, there will be individuals that spend the majority of their time in resource-poor habitat and others spending the majority of their time in resource-rich habitats and the mix of these two different dispersal distances will result in relatively high kurtosis. By contrast when the landscape is relatively uniform (either all patches are resource poor or all patches are resource rich), then there is little variation between individuals in their dispersal distances, so kurtosis is relatively low. This pattern is typically observed in real landscapes, where many butterflies have small dispersal distances but a few travel a great deal further (Schneider et al. 2003b). The differences between the models for kurtosis (Fig. 5; Fig. S2) demonstrate that such movement patterns are likely better represented if the interaction between behaviour and landscape composition is more fully taken into account by including variation both in the movement rate and activity.

A limitation of our study is that the responses of butterflies to habitat edges were not explored, as the focus was on changing time budgets and movement rates. Edge effects are important in butterfly movement, as they may cause individuals to cross boundaries less frequently (Haddad 1999; Conradt and Roper 2006; Schultz et al. 2012; Mair et al. 2015). Despite this, our dispersal predictions for the activity budget model are similar in range to those observed for *M. jurtina* across varying habitat types measured with mark–release–recapture (45–398 m) (Brakefield 1982a; Dover et al. 1992; Lörtscher 1997; Ouin 2000; Schneider et al. 2003a). These measures are notably affected by the size of the study area (Schneider 2003) and, consequently, the lowest of those estimates may result from small study sites influenced by edge effects not included in our model. It would be beneficial to include edge effects in future work studying dispersal in landscapes consisting of small and fragmented habitat patches and these movement rules can be incorporated in IBMs (Grant et al. 2018; Evans et al. 2019b). An additional limitation of our study is the simple dichotomy between resource-rich and resource-poor habitat, to understand how butterflies respond to a gradient of resource levels may require a better understanding of how motivation and perception interact to drive changes in local movements. This is achievable in butterflies, as energy budget models can track resource use and motivation (Evans et al. 2019b) and the host and nectar plants used by butterflies are generally well known (Dennis 1992). The generality of these processes suggests that the models including realistic mechanisms may provide improved movement forecasting for many species across changing landscapes.

## Electronic supplementary material

Below is the link to the electronic supplementary material.

Supplementary file1 (DOCX 1838 kb)
