# Supplementary material for: The importance of including habitat-specific behaviour in models of butterfly movement
Source: Oecologia. 2020 Apr 6;193(2):249–59. doi: 10.1007/s00442-020-04638-4 (PMC7320960; doi:10.1007/s00442-020-04638-4)
Supplement: Supplementary file 1 — Supplementary file1 (DOCX 1838 kb) [file 442_2020_4638_MOESM1_ESM.docx]

**Supplementary material 1**

Here we present the results of statistical analyses of the data presented in Figures 2, 3 and 4, and additional simulation output. Tables S1, S2 and S3 analyse the effects of habitat type and sex on the observational data presented in Figures 2 and 3. Figure S1 shows the effect of model structure on movement statistics for male butterflies and Figure S2 the effect of model structure, aggregation and the proportion of resource-rich habitat on movement statistics for female butterflies.

Table S1. Results of mixed effect linear models showing the effects of sex and habitat type on the components of movement, behaviour and 10-minute displacements. These effects are illustrated in Fig. 2. A random intercept of day of observation was introduced to control for variation between sampling days (σ = 0.1, 0.18, 0.37, 0.33). Standard error is shown in parentheses. A Bonferroni correction was applied to correct for multiple comparisons *p<0.05; **p<0.01; ***p<0.001

|  | Log Step speed  (m/s) | Log flight duration  (s) | Log Inter-flight duration (s) | Log Displacement rate (m/s) |
| --- | --- | --- | --- | --- |
| Sex (M) | -0.39***  (0.08) | 1.40***  (0.11) | -1.58***  (0.20) | 1.11***  (0.17) |
| Habitat type  (resource-poor)  Intercept | 0.74***  (0.08)  -0.64***  (0.08) | 0.45***  (0.10)  1.37***  (0.11) | -0.75***  (0.17)  4.82***  (0.19) | 1.46***  (0.15)  -3.93***  (0.17) |
| Df | 173 | 185 | 189 | 199 |
| R²(m) | 0.42 | 0.51 | 0.33 | 0.41 |
| R²(c) | 0.45 | 0.54 | 0.39 | 0.46 |

Table S2. Results of Wall-Raff rank-sum tests of angular distance showing the effects of sex and habitat type on turning angle. Bonferroni correction was applied to control for multiple testing. Significant effects are displayed in bold.

| V1 | V2 | χ² | p-value |
| --- | --- | --- | --- |
| Male resource-poor | Male resource-rich | 3.78 | 0.21 |
| Female resource-poor | Female resource-rich | 10.90 | **0.0039** |
| Male resource-poor | Female resource-por | 0.10 | 0.24 |
| Male resource-rich | Female resource-rich | 9.35 | **0.0089** |

Table S3. Results of Dirichlet regression showing the effects of sex and habitat type on the proportion of time spent in each behaviour. These effects are illustrated in Fig. 3. Standard error is shown in parentheses. A logistic regression was used to predict oviposition across the two habitat types. Bonferroni correction applied to p-values *p<0.05; **p<0.01; ***p<0.001

|  | Nectaring | Flight | Inactive | Basking | Oviposition  *GLM* |
| --- | --- | --- | --- | --- | --- |
| Sex (M) | -0.011  (0.14) | 1.26***  (0.15) | -0.26  (0.16) | 0.21  (0.14) | X |
| Habitat type (resource-poor)  Intercept | -0.56**  (0.14)  1.17***  (0.13) | 0.602**  (0.15)  -1.39***  (0.13) | 0.55**  (0.16)  -0.56***  (0.13) | 0.01  (0.14)  -1.75***  (0.12) | 2.05**  (0.59)  -2.35***  (0.52) |
| N | 200 | 200 | 200 | 200 | 99 |


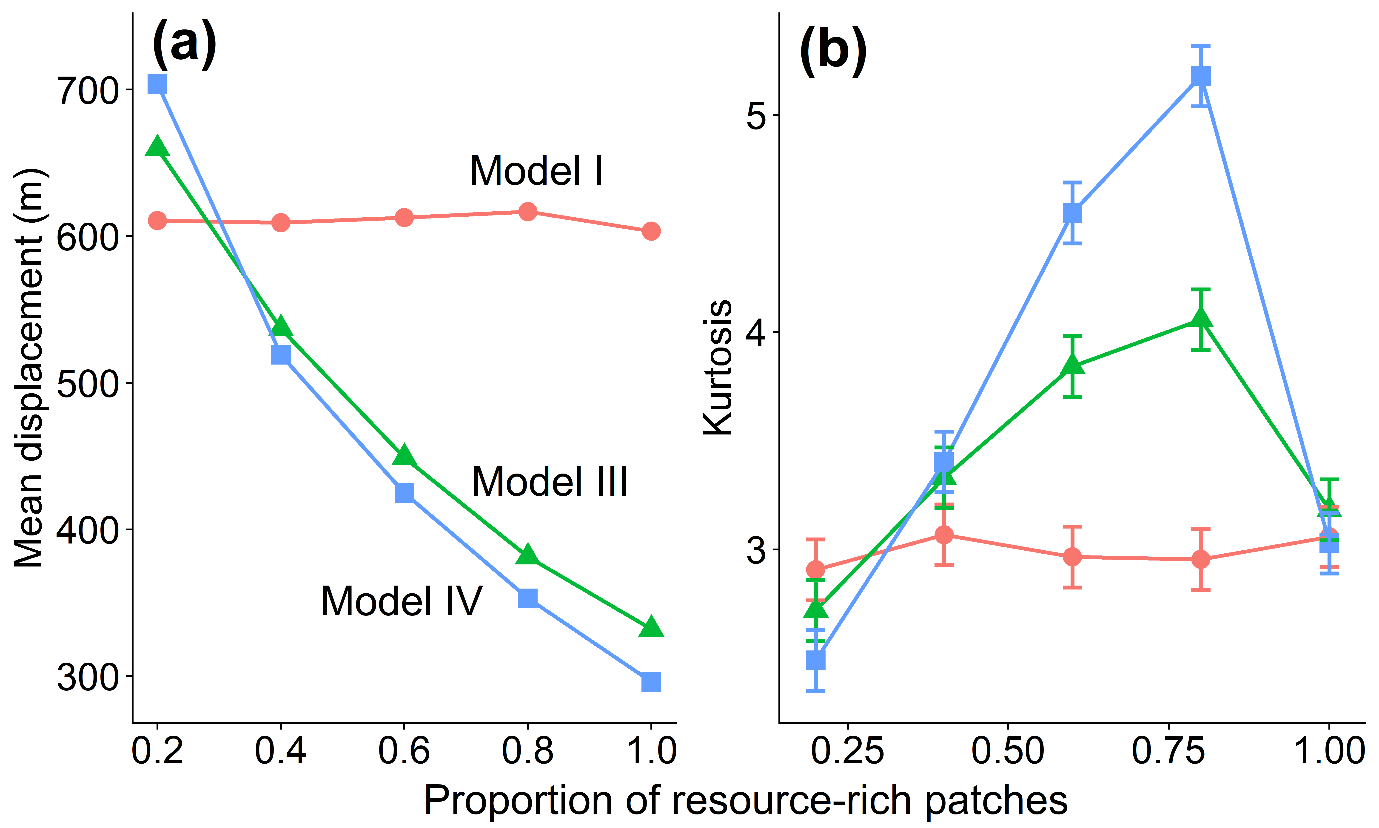


Figure S1. To supplement Fig. 5 we here show the effect of model structure on five-day displacement statistics for male butterflies. a) Mean displacement; b) Kurtosis. As in Fig. 5 Model I represents a null model, without habitat-specific movement and behaviour; Model III represents the standard movement model with habitat-specific steps and turns and Model IV represents the full movement model, where both movement rules and motivation to move are all habitat-specific. 95% confidence intervals for kurtosis were estimated as ± 0.14 (Wright and Herrington 2011)


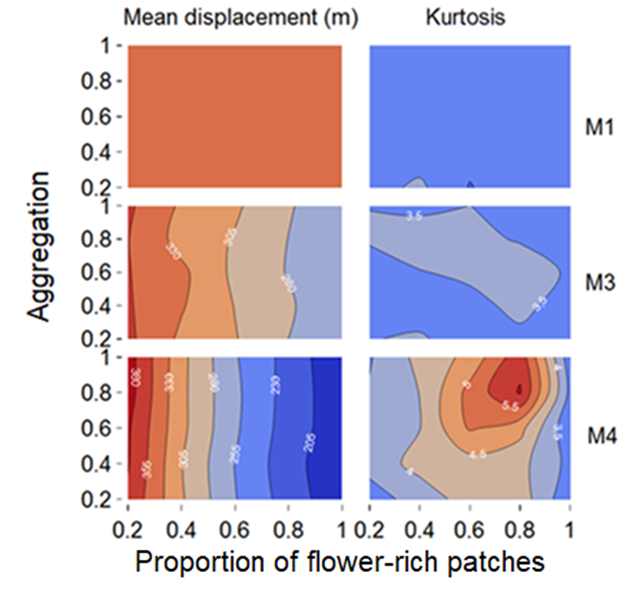


Figure S2. The effect of model structure, aggregation and the proportion of resource-rich habitat on movement statistics for female butterflies. The model type is shown on the right hand side of the panels. Model 1 (M1) represents a null model, without habitat-specific movement and behaviour; Model 3 represents the standard movement model with habitat-specific steps and turns and Model 4 represents the full movement model

**Supplementary material 2: ODD butterfly movement model**

*Purpose*

The purpose of the IBM is to quantify how activity budgets and movement capacity influence the displacement rate of butterflies across heterogeneous landscapes. This is achieved by using habitat-specific movement and activity parameters to explicitly simulate the transitions between flight and inter-flight that occur during butterfly movement. Here, displacement rate is the Euclidean distance divided by the movement time and habitat heterogeneity is incorporated as a simple dichotomy between resource-poor and resource-rich patches.

*Entities, state variables and scales*

The model landscape consists of two-dimensional 20m² patches that are either resource-poor or resource-rich. The total size of the landscape was 3 x 3 km. The categorisation of the patches is controlled using a fractal mid-point displacement algorithm (Saupe, 1988) developed for NetLogo in Jackson and Fahrig (2012). This generates realistic natural distributions of heterogeneous landscapes. The landscape was toroidal and of sufficient size that displacement by individuals was always the shortest Euclidean distance between their starting and ending locations on the toroid. Butterflies move across this landscape and are described by their sex and spatial location. The model proceeds in one-second time-steps.

*Process overview and scheduling*

*Submodels*

Further detail on the development of submodels is given below. Where data below are drawn from field observations these are presented in the main text.

1. *Habitat recognition.* Butterflies determine whether their current location is in a resource-poor or resource-rich patch. This subsequently affects processes in *flight* and *inter-flight*.

2. *Activity selection.* Butterflies select their next activity, which cycles between periods of flight or inter-flight. The duration of flights and inter-flights is drawn from distributions of those observed in the field and is habitat-specific.

3. *Inter-flight.* Between flights, butterflies remain stationary.

4. *Flight.* Butterflies move across the landscape according to a correlated random walk until the current flight time has elapsed. The rate of movement and the straightness of flight is habitat-specific and is drawn from distributions collected from field observations.

*Initialisation*

Population size set on initialisation. Butterflies are located randomly.

*Design concepts*

*Basic principles*

Displacement is a consequence of habitat-specific movement rates and activity of butterflies within heterogeneous landscapes. Movements are modelled using a modified correlated random walk that incorporates the habitat-specific changes in activity budgets and movement rates. Interaction with the environment is achieved through the butterflies sensing their current location.

*Emergence*

The main feature emerging from the model is individual displacement distances in response to a given habitat structure. The quantity and organisation of resource-rich and resource-poor habitat within the landscape affects the displacement of butterflies. As the locations experienced by the butterflies differ, this affects the kurtosis of the combined displacement distribution. Finally, habitat-specific movement and activity affect the density of butterflies within the different habitat types.

*Adaptation*

Movement rates and activity change in response to habitat type that increases time spent in resource-rich habitat and reduces time in resource-poor habitat.

*Sensing*

Butterflies can perceive the quality of their current patch.

*Stochasticity*

All movement in the model is stochastic as butterflies draw randomly from distributions representing *flights*, *inter-flights*, *step distances* and *turning angles.*

*Sub-model details*

1. *Habitat recognition*

Butterflies check the identity of the current patch at every time-step during the simulation. The current location determines from which distribution the duration of the next flight is drawn, the rate of movement during the flight, and the subsequent inter-flight period.

1. *Activity selection*

Activity is modelled by drawing from behavioural data collected on *M. jurtina* activity-budgets in flower-rich and flower-poor areas. On initialisation 20000 draws from the observed flight and inter-flight durations distributions of both habitat types are imported into the model. In *activity selection,* the butterflies cycle between drawing durations of either *flight* or *inter-flight*. At the start of the simulation, the butterflies first select an inter-flight duration and the *inter-flight* procedure runs for the selected number of time-steps. Once this period has elapsed a flight duration is then called and the *flight* procedure runs in the same manner. The process repeats for the duration of the simulation.

1. *Inter-flight*

After being selected by *activity selection* butterflies in inter-flight remain stationary.

1. *Flight*

Butterflies move during flight through a correlated random walk with steps and turns derived from draws of the observed distributions measured across the habitat types. To move individuals draw step distances from habitat-specific distributions of step lengths observed for flights of that duration. For example, if a four second flight was drawn, a step from the distribution of step lengths observed after four second flights would be selected. The butterfly then moves forward at a rate such that the step length is completed in this the flight time (speed = step distance / step duration). As step lengths were measured up to a maximum of every 15 seconds a long flight may result in multiple steps being drawn before the flight has been completed. At the end of each step and flight, a new heading is drawn from a habitat-specific distribution and added to the current heading. Turning angles are drawn from a Von-Mises distributions fitted to the turning angle data in both habitats.

**Supplementary material 3: site map**


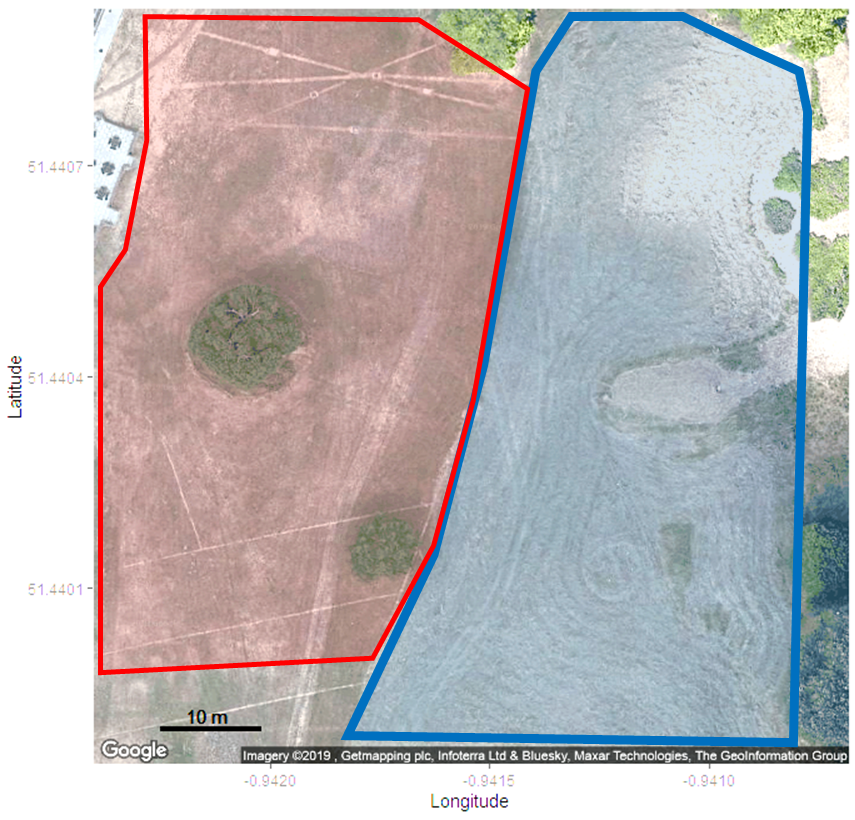


Figure S3. Main study site from fieldwork. The blue section shows a region of the meadow grassland (after the grass had been mown at the end of the summer) and the red mown grass. Scale bar is shown in black.
